# Interfacial Dynamics in the Fabrication of Various Concave Hydrogel Discs for Enhanced Biosensing

**DOI:** 10.3390/polym17172341

**Published:** 2025-08-28

**Authors:** Amin Ghaffarzadeh Bakhshayesh, Kara Cook, Huiyan Li

**Affiliations:** School of Engineering, University of Guelph, Guelph, ON N1G 2W1, Canada; amingb@uoguelph.ca (A.G.B.); kcook08@uoguelph.ca (K.C.)

**Keywords:** three-phase flow modeling, biosensing, concave hydrogel discs, interfacial dynamics

## Abstract

Hydrogel-based biosensors are commonly used in diagnostic applications. However, their performance remains constrained by slow analyte diffusion within polymer matrices, particularly when larger biomolecules are involved. Concave hydrogel geometries present a promising solution to enhance diffusion rates through increased surface area. However, the interfacial dynamics governing their formation must be studied. In this research, we investigated the interfacial dynamics that influence the formation of concave hydrogel discs fabricated by a simple pipetting method. We characterized the fluid interactions occurring during droplet deposition of alginate and CaCl_2_ solutions. A three-phase flow model incorporating confocal microscopy validation was employed to simulate time-dependent interfacial behaviors. Concave hydrogel discs fabricated with alginate-first deposition exhibited 83% larger surface area compared to hemispherical counterparts at a CaCl_2_: alginate volume ratio of one. Increasing the volume ratio further enhanced both surface area and diameter, though this highlighted limitations for microscopy-based detection. According to our results, reaction speed in alginate concave hydrogel discs can be controlled by varying the volume of CaCl_2_ solution while keeping the volume of alginate solution constant, which changes the surface area while maintaining constant hydrogel volume.

## 1. Introduction

Hydrogels consist of three-dimensional networks of cross-linked polymers that can absorb and retain significant volumes of water. Biocompatible and electrically conductive hydrogels are particularly attractive for biomedical applications, including biosensing [1]. These hydrophilic, cross-linked macromolecular polymers can retain up to 99% of their mass as water or biological fluids [2,3]. Hydrogel-based sensors offer notable advantages in diagnostic technologies due to their porous structure, which allows for the integration of numerous biomolecular recognition elements within a highly aqueous and biocompatible environment [4,5]. The hydrated structure of hydrogels also effectively reduces nonspecific biomolecule adsorption, significantly decreasing background signal interference. This effect arises from the polymers’ antifouling properties and the solution-like milieu provided by the hydrogel. Consequently, biomolecules that are not chemically addressed will not bind to the surface [4]. For instance, polyacrylamide-based synthetic hydrogels were utilized in immunoassays due to their low nonspecific absorption [6]. Additionally, hydrogels facilitate precise modulation of sensing molecules and easy integration with transducing components [4].

However, molecular mass transfer remains a significant challenge in receptor-based biosensors, as slow analyte diffusion prolongs assay times [7]. In micromixers, an increased interfacial area enhances diffusion between solutions; indeed, this surface area enhancement allows for a higher mixing index at the micromixer’s outlet with lower Reynolds numbers than can be achieved with a smaller interfacial area even at higher Reynolds numbers [8]. In hydrogel-based systems, sensitivity is similarly limited by the slow diffusion rates of larger biomolecules, such as antibodies, within the hydrogel matrix [9,10]. Researchers are actively exploring strategies to enhance diffusion within hydrogels, particularly through shape modification and increased surface-to-volume ratios that minimize diffusion distances. As the surface-area-to-volume ratio increases, a greater number of substrate molecules per unit hydrogel mass becomes accessible to bioparticles; the initial reaction rate increases, and the sensor responds more rapidly [11]. Several advanced fabrication methods have been employed to this end. For instance, Montero et al. demonstrated that 100 nm hydrogel films exhibited superior performance compared to thicker 200 nm films, owing to faster hydration and improved analyte diffusion [12]. In a different fabrication scheme, Mandon et al. employed digital light processing (DLP) 3D printing to fabricate propeller-shaped hydrogel sensors, achieving increased signal strength [13]. In another instance, An et al. developed a vortex ring freezing technique enabling the large-scale production of uniquely shaped hydrogel particles, including teardrops, jellyfish, and donuts [14]. In this method, dropping a droplet into a miscible liquid is an easy method of creating a vortex ring. Upon striking the free surface of a miscible liquid with sufficient impact speed, the droplet deforms and curls back on itself to dissipate its energy. As the edge continues this curling motion, the center of the droplet becomes progressively thinner. Eventually, the center grows too thin to bear surface tension; it then breaks, forming a donut shape. Drop deformation on impact is resisted by interfacial tension. Following immersion, the rate of hydrogel formation is determined by the speed of fluid mixing compared to the speed of reaction. In this method, vortex-ring-derived particle formation occurs when a nanoclay solution droplet is introduced into a crosslinking buffer pool. These particles, benefiting from improved surface-to-volume ratios, show significant potential for bioencapsulation and cell culture applications [14].

**Figure 1:**
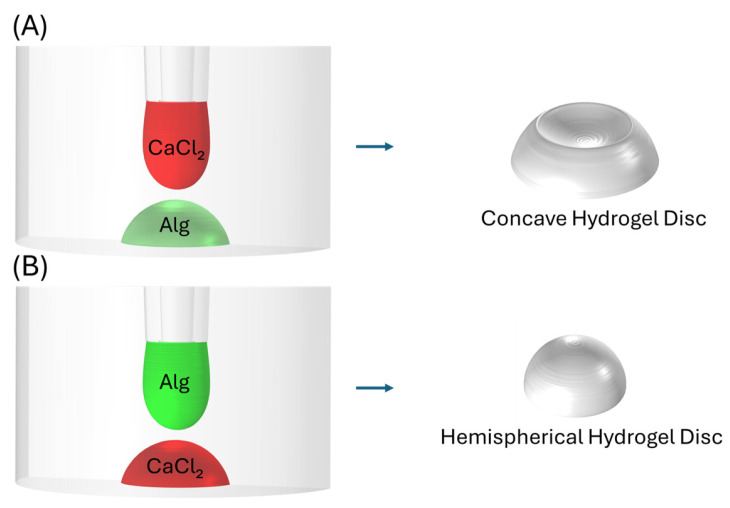
Schematic of the hydrogel disc preparation: (**A**) concave hydrogel discs; (**B**) hemispherical hydrogel discs.

Another notable example of optimizing surface-to-volume ratios includes stop-flow lithography, used to produce porous, nonspherical hydrogel particles with tunable pore sizes [15]. A highly enlarged surface area, provided by nonspherical particles and their discrete interconnected microstructures, can broaden the range of swelling-dependent opacity color shift, as these features permit facile diffusion of the solvent within the structures. Nonspherical particles can afford greater capacity for drug loading, as their geometry increases the effective core volume. Taken together, these properties make these nonspherical hydrogel particles suitable candidates for drug loading and optical sensing applications. While these methods offer high precision and control over particle geometry, they often require specialized equipment and complex microfabrication facilities. In contrast to these complex methods, a straightforward and highly accessible technique to increase hydrogel surface area was developed by our group, which relies on a simple pipetting process to form concave hydrogel discs [16]. This technique, requiring no complex microfabrication facilities, relies on the interaction between droplets of CaCl_2_ and alginate (Alg) deposited sequentially onto a microscope slide. Figure 1A illustrates the spotting order necessary to create a concave shape. The resulting concavity emerges from the downward momentum of the upper droplet and the corresponding downward progression of crosslinking, given that alginate lies beneath CaCl_2_. Conversely, reversing the droplet spotting order (Figure 1B) yields hemispherical hydrogel discs due to upward crosslinking.

While advanced techniques like 3D printing offer high precision, simpler deposition-based methods remain attractive due to scalability and cost-efficiency. However, there remains a gap in accurate modeling of concave hydrogel discs. Precise analysis of interfacial dynamics is essential for accurately determining surface area and volume, thereby optimizing the shape of hydrogel discs to enhance assay speed and signal intensity. Addressing this gap, the current study provides a detailed numerical and experimental examination of the interfacial phenomena governing the formation of concave and hemispherical alginate hydrogel discs. The analysis of interfacial dynamics in our method is novel, as it differs fundamentally from vortex ring freezing methods. Here, a crosslinker droplet impacts a confined volume of polymer solution (alginate), with the droplet maintaining relatively low impact velocity throughout its descent. The objective of this work is to understand these interfacial dynamics, highlighting the advantages of concave geometry, particularly increased surface area, in biosensing applications. For this purpose, a three-phase flow model was developed, incorporating confocal microscopy images of hydrogels to simulate time-dependent interfacial behaviors numerically. The surface areas of hydrogel discs were numerically calculated, and the impact of varying volume ratios on the dimensions and surface area of concave hydrogel discs was numerically investigated.

## 2. Materials and Methods

### 2.1. Materials

Alginic acid sodium salt from brown algae (low viscosity) came from Sigma-Aldrich (Saint Louis, MO, USA, Catalog No. A1112). The polymer has a molecular weight (MW) of 100,000 g/mol and contains approximately 61% mannuronic acid and 39% guluronic acid, which gives an M/G ratio of 1.56 [17]. Milli-Q water with a resistivity of 18.2 MΩ·cm at 25 °C was used for all aqueous solutions. Glycerol was sourced from Sigma-Aldrich (Saint Louis, MO, USA, Catalog No. G5516). Invitrogen Dynabeads Protein A (2.8 µm diameter, 30 mg/mL; Catalog No. 10001D) was obtained from Thermo Fisher Scientific (Pittsburgh, PA, USA). Fluorescein (FITC)-conjugated rabbit anti-goat IgG(H+L) antibody arrived from Proteintech (Rosemont, IL, USA, Catalog No. SA00003-4). Phosphate-buffered saline (1× PBS) served as the dilution buffer. Calcium chloride (CaCl_2_, anhydrous powder) was obtained from Sigma-Aldrich (Saint Louis, MO, USA, Catalog No. C4901). Ethylenediaminetetraacetic acid disodium salt dihydrate (EDTA) came from Fisher Chemical (Catalog No. S311). For hydrogel fabrication and imaging, standard polystyrene Petri dishes and polystyrene microscope slides were used. A rectangular magnet (12 mm × 6 mm × 2 mm) was employed for the dissolution of hydrogel discs.

### 2.2. Methods

#### 2.2.1. Experimental Methods

##### Solution Preparation and Hydrogel Fabrication

A 2 wt% alginate solution was prepared by dissolving 0.1 g of alginic acid sodium salt from brown algae into 5 mL of Milli-Q water. The mixture was stirred overnight at room temperature to ensure complete dissolution. Glycerol was then added to achieve a final glycerol concentration of 20% by volume. Glycerol is usually included in the printing buffer to limit evaporation [18]. Invitrogen Dynabeads Protein A were resuspended by vortexing for 30 s, and subsequently, 20 µL of this suspension was transferred into a fresh tube. For 3D imaging of hydrogels to validate numerical studies via confocal microscopy, fluorescently labeled secondary antibodies were incorporated. For this purpose, 1 µL of Fluorescein (FITC)-conjugated rabbit anti-goat IgG(H+L) was diluted in 49 µL of 1× PBS.

The tube containing Dynabeads was placed on a magnet to separate beads from the solution, after which the supernatant was carefully removed. Following removal from the magnet, the FITC-rabbit anti-goat IgG PBS solution was added directly to the beads. The mixture was gently mixed and rotated for 30 min in the dark. The tube was then placed again on a magnet to separate Dynabeads from the solution, and the supernatant was removed. Subsequently, 5 µL of Dynabeads suspension was combined with an equal volume of alginate solution, forming the Dynabeads–alginate mixture (alginate solution).

Using a micropipette, 0.5 µL droplets of this mixture were deposited onto a moist polystyrene Petri dish, as illustrated in Figure 1A. To form concave hydrogels, droplets ranging from 0.25 to 1.5 µL of 0.1 M calcium chloride solution were added onto each Dynabeads–alginate spot. For the fabrication of hemispherical hydrogel structures, 0.5 µL of the Dynabeads–alginate mixture was precisely deposited onto pre-formed 0.1 M CaCl_2_ droplets, as depicted in Figure 1B. Immediately afterward, Petri dishes were placed in the confocal microscope to minimize errors in hydrogel thickness measurement caused by evaporation.

##### Microscopic Imaging

Confocal imaging was performed using a STELLARIS 5 confocal microscope (Leica Microsystems GmbH, Wetzlar, Germany). To enhance accuracy in hydrogel thickness measurements, an upright configuration was chosen, since inverted configurations require the laser to pass through a polystyrene Petri dish, potentially introducing refraction errors. For conventional microscopic imaging, standard polystyrene microscope slides were employed. Images were captured with a Nikon Eclipse Ti microscope (Nikon Corporation, Tokyo, Japan) equipped with a Nikon DS-Qi1Mc monochrome digital camera (Nikon Corporation, Tokyo, Japan) and a 4× lens.

##### Hydrogel Dissolution Assay

To examine the dissolution rate of concave and hemispherical magnetic hydrogel discs, a rectangular magnet was employed. The dissolution rate of hydrogel discs represents a key functional improvement for biosensing workflows, particularly for bioassays that require the release of captured analytes for subsequent downstream analysis [19]. This rapid dissolution shortens the workflow and reduces the overall assay time. After the hydrogel discs were formed and crosslinked for 20 s, they were washed three times with 3 µL of Milli-Q water and then positioned 2 mm from the rectangular magnet. Subsequently, 3 µL of a 0.1 M EDTA was dispensed onto each disc. A camera recorded the dissolution of the discs along with the movement of Dynabeads as they were drawn toward the magnet. To enhance contrast between the inner core and the outer ring of the hydrogel discs, the Dynabead concentration in this experiment was reduced two-fold relative to the initial alginate solution.

#### 2.2.2. Numerical Methods

To predict the final geometry of the hydrogel, we performed direct simulations of a three-phase flow system. This numerical model integrates laminar flow dynamics with the Ternary Phase Field interface, implemented within the COMSOL Multiphysics 6.3 software. Designed as a time-dependent simulation, it models the behavior of a falling droplet from a pipette tip onto another droplet resting on a solid substrate. The simulation ran from 0 to 0.5 s, using a time step of 0.0002 s.

**Figure 2:**
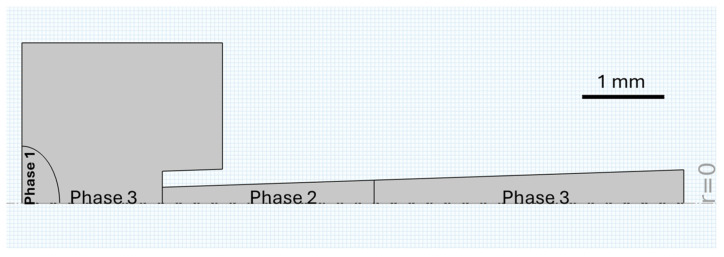
Two-dimensional axisymmetric geometry of the numerical model.

Multiple simulations were developed, each representing a pipette tip loaded with varying volumes of CaCl_2_ solution and a fixed volume (0.5 μL) of alginate solution. The remaining space within the pipette tip was modeled as air. To maintain laminar flow conditions, the inlet velocity of air at the input of the pipette tip was set to 0.5 m/s. The geometric parameters of the pipette tip were based on measurements from a Fisherbrand 02-707-149 micro-pipette tip, as shown in Figure 2.

The target droplet, deposited on a horizontal surface, was modeled as a sessile droplet with a semi-elliptical shape defined by semi-axes of 0.47 mm and 0.712 mm. A vertical gap of 1.28 mm separated the pipette tip from the droplet. Given the inherent symmetry of both the pipette tip and the droplet, we employed a 2D axisymmetric geometry to reduce computational cost. Three-dimensional results were reconstructed by revolving the 2D dataset around the axis of symmetry. This approach enabled efficient yet accurate modeling without compromising the accuracy of the flow dynamics.

In addition to the airflow inside the pipette, which imitates manual pipetting, gravitational acceleration was applied uniformly across all domains. The simulated system comprises three immiscible phases as illustrated in Figure 2.

Material properties were defined based on available literature and experimental measurements. At 20 °C and 1 atm, the density and dynamic viscosity of air were set to 1.205 kg/m^3^ and 18.21 × 10^−6^ Pa·s, respectively [20]. The dynamic viscosity of a 0.1 mol·kg^−1^ CaCl_2_ solution was set to 1.0319 × 10^−3^ Pa·s [21]. The alginate solution, measured at 25 °C and under a shear rate of 0.5 s^−1^, had a dynamic viscosity of 49.5 Pa·s [22]. A shear rate of 0.5 s^−1^ was chosen because of the droplet’s low impact velocity during fall. We measured the densities of the alginate and CaCl_2_ solutions at 21 °C and found them to be 1092 kg/m^3^ and 1018 kg/m^3^, respectively.

Surface and interfacial tensions play a critical role in our phase-field model. The surface tension values for the 0.1 mol·kg^−1^ CaCl_2_ and alginate solutions were taken as 0.0878 N/m and 0.0532 N/m, respectively [22,23]. However, the interfacial tension between the two liquids remains unknown. While the Girifalco–Good equation provides a theoretical route to estimate this parameter [24], it requires a dimensionless interaction constant that has not been established in this context.

Contact angles further influence wetting behavior on solid surfaces. The contact angle of water on polystyrene surfaces ranges from 72° to 86° [25,26]. For polypropylene (the material used in the pipette tips), reported water contact angles lie between 85° and 96° [25]. However, direct measurements of contact angles for both CaCl_2_ and alginate solutions on polystyrene and polypropylene substrates were unavailable. Similarly, the contact angle at the CaCl_2_–alginate interface on the solid surface was not previously defined.

To find unknown variables, we experimentally determined the contact angles of both solutions on relevant substrates, as presented in the results section. These values, in turn, allowed us to infer the unknown interfacial tensions by comparing simulation outputs with confocal microscopy images of the formed hydrogel discs. Following parameter calibration and validation, the numerical model was used to examine the spatial configuration of the alginate solution phase.

Finally, the surface area of each phase at various time points was quantified by computing surface integrals over isosurfaces corresponding to individual phases. This enabled detailed assessment of phase distribution dynamics throughout the simulation domain.

## 3. Results

### 3.1. Experimental Results

To calculate the exact surface area of each hydrogel disc, we developed a numerical method using experimental data. Contact angles of prepared solutions on Petri dish slides and pipette tips made of polypropylene remain unreported in the literature. We therefore measured these values by capturing images of sessile drops on polystyrene slides and photographing pipette tips with solutions.

**Figure 3:**
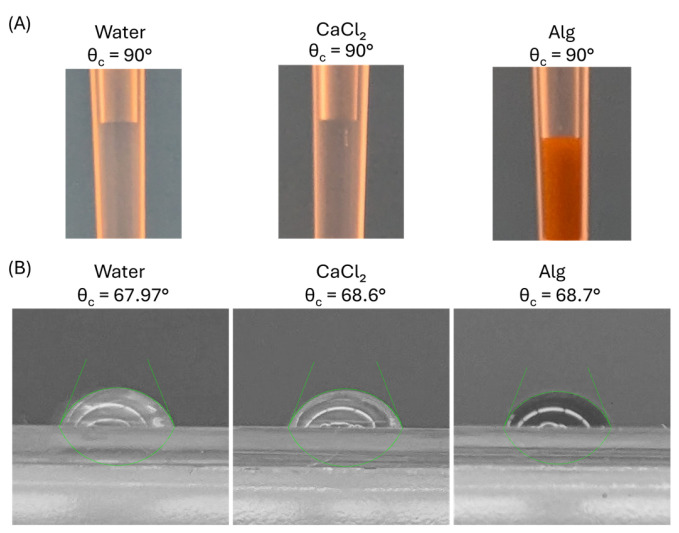
Contact angle measurements of water, CaCl_2_, and alginate solution on polypropylene tips (**A**) and polystyrene (**B**) surfaces.

Contact angle measurements of solutions on pipette tip surfaces revealed similar values for all tested solutions, including water, 0.1 M CaCl_2_, and alginate solution, as depicted in Figure 3A. These angles approximated 90°, consistent with the reported range for water on polypropylene (85° to 96°) [25].

For contact angle calculations of alginate solution and 0.1 M CaCl_2_ solution drops on polystyrene slides, we employed the LB-ADSA plugin of ImageJ 1.54g software [27]. The plugin requires black-and-white images, necessitating image conversion before analysis. Figure 3B demonstrated that contact angles of water and other solutions exhibit close similarity. Results indicated contact angles of 67.97°, 68.6°, and 68.7° for water, 0.1 M CaCl_2_, and alginate solution on polystyrene, respectively. Our measured values showed deviations of 5% below the reported literature range (72–86°) for water on polystyrene [25]. This deviation may result from surface preparation differences or measurement technique variations. The solutions’ contact angles closely matched those of water. This similarity can be attributed to the low concentration of alginic acid sodium salt and CaCl_2_ in water. Additionally, glycerol did not significantly alter solution surface tension because it lacked tensio-active properties [28].

**Figure 4:**
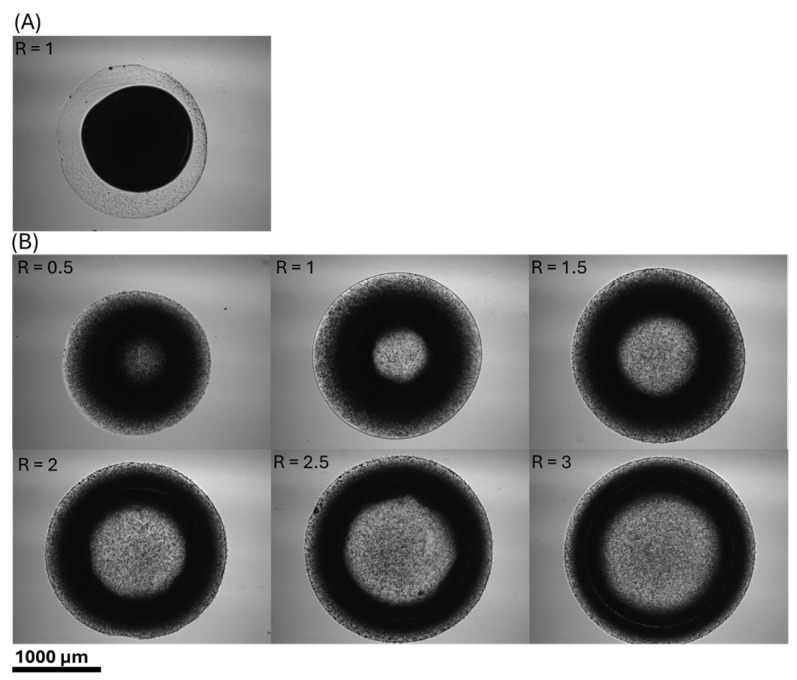
Microscopic images of hydrogel discs: (**A**) hemispherical hydrogel disc; (**B**) concave hydrogel disc.

Figure 4 illustrates concave and hemispherical hydrogel discs produced using the methods described in this paper. Images were captured using a Nikon Eclipse Ti microscope equipped with a Nikon DS-Qi1Mc monochrome digital camera and a 4× lens. Samples were spotted on polystyrene microscope slides. Figure 4A presents a hemispherical hydrogel disc formed when the volume ratio of 0.1 M CaCl_2_ to alginate solution equals 1 (R = 1). In every case, the volume of alginate remained constant at 0.5 µL. This configuration occurred when alginate solution was spotted after 0.1 M CaCl_2_. Figure 4B demonstrates concave hydrogel discs with varying volume ratios. The concave geometry created a brighter appearance at the disc center due to reduced thickness, which corresponded to a lower concentration of Dynabeads compared to the outer regions. Analysis revealed that increasing volume ratios correlated with proportional increases in both hydrogel disc diameter and inner circle diameter.

**Figure 5:**
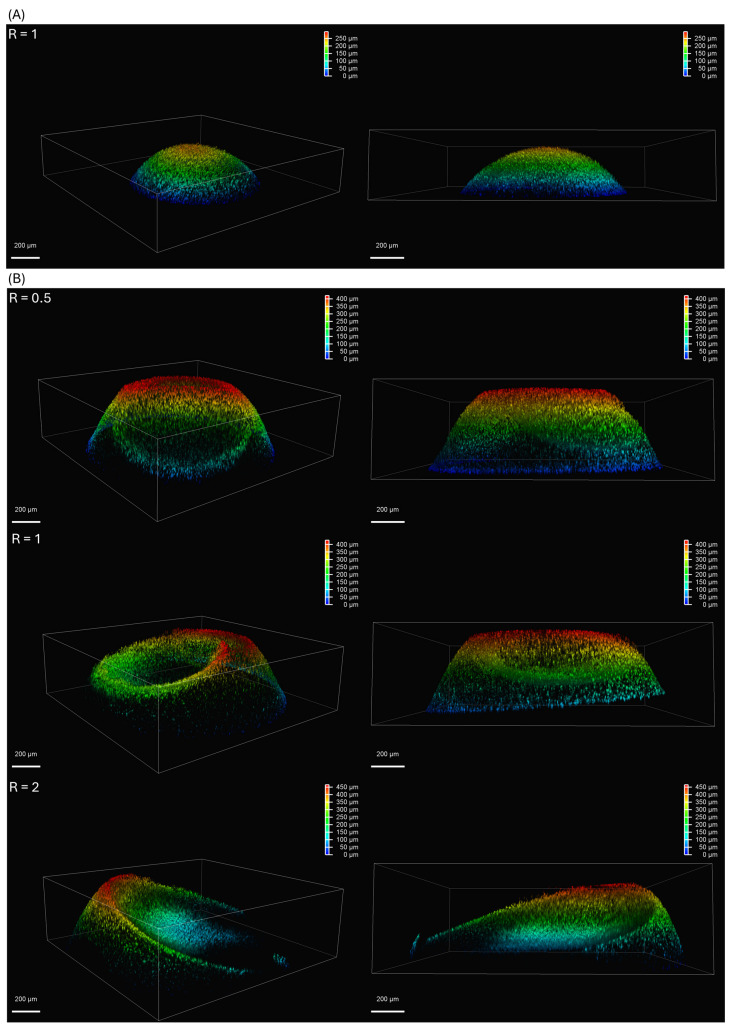
Confocal images of hydrogel discs: (**A**) hemispherical hydrogel disc; (**B**) concave hydrogel disc.

Figure 4 does not provide sufficient detail regarding the thickness of different hydrogel components. To obtain a three-dimensional perspective of the hydrogel structure, we utilized a STELLARIS 5 confocal microscope. Figure 5 presents the 3D confocal images of both hemispherical and concave hydrogel discs. We employed LAS X office software for post-processing, generating 3D images of the hydrogel discs from confocal image layers captured at 2.4 µm intervals of hydrogel thickness. Subsequently, depth coding and corresponding legends were incorporated into the images using LAS X office to enhance the visualization of the varying thickness across different parts of the hydrogel disc. To decrease drying and evaporation, samples were spotted onto a polystyrene Petri dish, which was used as a humid chamber. In all experiments, the alginate volume remained constant at 0.5 µL.

Figure 5A illustrates a hydrogel disc formed when the alginate solution was spotted following the application of 0.1 M CaCl_2_ (CaCl_2_-First). This configuration produced a hemispherical hydrogel disc. Conversely, Figure 5B displays the hydrogel’s shape when the alginate solution was spotted prior to the 0.1 M CaCl_2_ (alginate-first). This figure demonstrates the formation of a concave hydrogel for various volume ratios of 0.1 M CaCl_2_ to alginate solution. Our results indicated that increasing this volume ratio leads to an increase in the hydrogel disc’s diameter and a reduction in the thickness of the ring.

**Figure 6:**
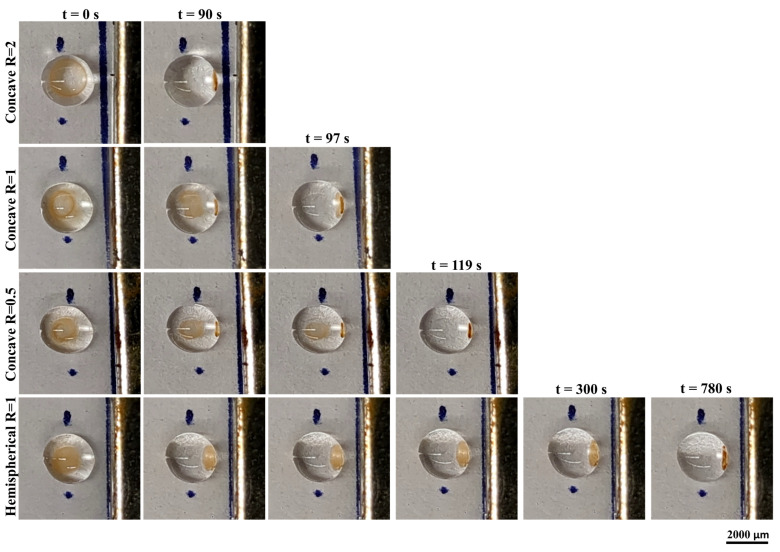
Dissolving rates of concave and hemispherical magnetic-responsive hydrogel discs in an EDTA solution over time.

In bioassays, dissolution of hydrogel enables release of captured analytes for downstream analysis. To experimentally compare dissolution of concave and hemispherical hydrogel discs, we first fabricated both geometries with encapsulated magnetic beads and then applied 0.1 M EDTA to dissolve the matrix. Figure 6 and Video S1 depict the dissolution behavior of magnetically responsive concave and hemispherical hydrogel discs in EDTA over time. For hemispherical discs, the process proceeded gradually and was essentially uniform. By contrast, concave discs displayed two phases. The initial phase resembled the hemispherical case: a uniform, layer-by-layer erosion. The second phase was a rapid collapse that occurred at a critical time point. This behavior reflects greater positional stability of the concave discs on the surface compared with the hemispherical counterparts. Owing to the hemispherical topology, shortly after EDTA was dispensed, the entire hydrogel shifted toward the magnet, whereas concave discs remained in place; uniform dissolution then caused material to accumulate on one side of the EDTA droplet. At a critical time, the entire concave hydrogel collapsed abruptly. According to Figure 6 and Video S1, the concave hydrogel disc with R = 2 collapsed at 90 s, R = 1 at 97 s, and R = 0.5 at 119 s, whereas dissolution of the hemispherical hydrogel discs required 780 s.

### 3.2. Validation

Once the contact angles were determined experimentally, we estimated the final unknown variable from confocal images. This process entailed simulating three-phase models built from the given and calculated variables. In the initial step, we estimated the interfacial tension between the two solutions by calibrating our numerical model with confocal images of concave hydrogels where R = 1. An interfacial tension of 0.0747 N/m yielded errors between the dimensions from confocal images and the alginate phase in the numerical model that ranged from 0.95% to 6.21%. Using this same interfacial tension value, we developed numerical models for all confocal images. A summary of numerical (N) versus experimental (E) dimensions, along with associated errors, is provided in Table 1.

During the experimental procedure, the difficulty of spotting the second droplet precisely onto the center of the first using a pipette resulted in non-uniform ring thickness in the concave hydrogels. For this reason, we used the average ring thickness in Table 1 for comparison against the numerical results. For the hemispherical hydrogel discs, maximum height was used as the comparative metric instead of ring thickness. According to Table 1, the errors were within a reasonable range, with two notable exceptions. The first was the ring thickness for the concave hydrogel disc when R = 0.5, where the error between experimental and numerical results was 24.85%. As illustrated in Figure 5, the thickness of this hydrogel was slightly outside the scanning range of the confocal microscope, suggesting the real thickness was likely greater than what was measured.

**Figure 7:**
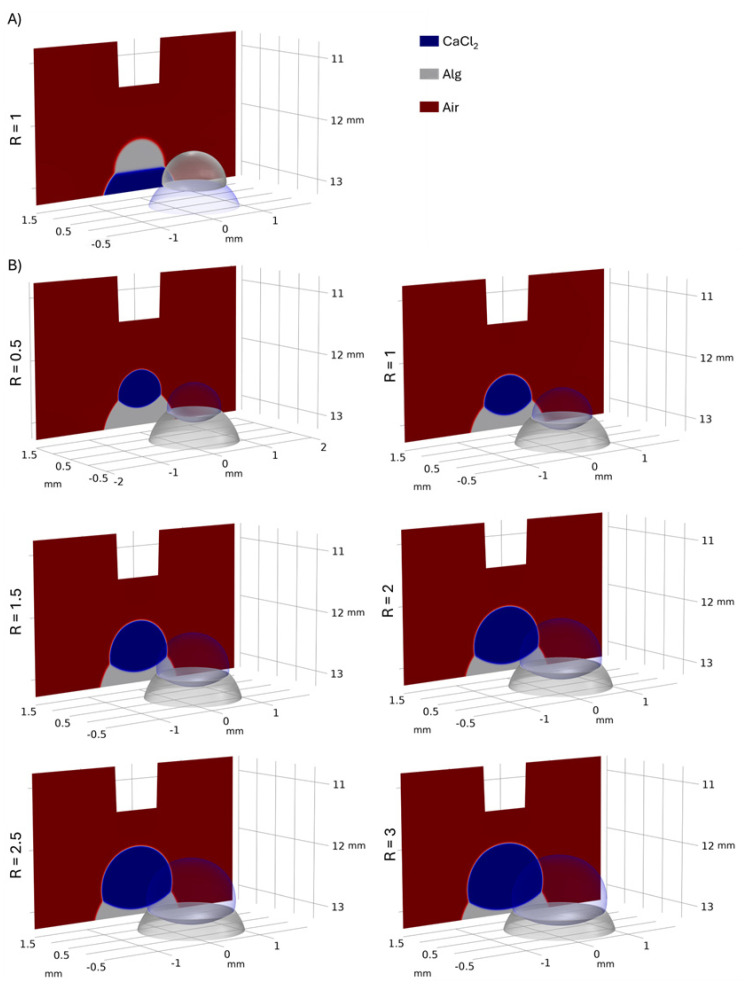
Numerical model of the droplet interactions: (**A**) CaCl_2_-first; (**B**) alginate-first.

The second discrepancy was the maximum height of the hemispherical hydrogel disc at R = 1, which showed an error of 35.55%. Based on direct observation and numerical results presented in Figure 7, distinct spatial arrangements emerged depending on the order of solution deposition. When alginate solution was spotted prior to 0.1 M CaCl_2_ application, the CaCl_2_ solution covered both the alginate solution and the resulting hydrogel. Conversely, when alginate solution was applied subsequent to 0.1 M CaCl_2_ deposition, both the alginate solution and the final hydrogel remained positioned atop the CaCl_2_ layer. In the first case, the CaCl_2_ layer prevented the hydrogel from drying; consequently, its thickness changed slowly compared to the second case during confocal imaging.

Although the interfacial tension between 0.1 M CaCl_2_ and alginate is dynamic during crosslinking, these numerical results showed that assuming an average interfacial tension allows for a reasonable prediction of the final hydrogel geometry. Given that both experimental and numerical results consistently showed that the solutions’ interfacial area did not interact with the walls, there was no need to define the contact angle at the CaCl_2_–alginate interface on the solid surface.

### 3.3. Numerical Results

Figure 7 presents both 3D and 2D visualizations of the interactions between 0.1 M CaCl_2_ and alginate solutions, demonstrating the impact of deposition order and volume ratios (R). This figure reveals a distinct difference. Unlike the alginate-first configuration, the interfacial area remained flat when alginate was spotted after CaCl_2_. Given the minimal density difference between the 0.1 M CaCl_2_ and alginate solutions, this interfacial shape was mainly attributed to differences in their dynamic viscosities.

The simulated shape of the alginate phase closely corresponded to the experimentally observed hydrogel shapes. This alignment indicated that when droplets gently interacted, immediate crosslinking at the interface generated sufficient interfacial tension, thereby preventing the droplets from mixing. This finding validated the initial assumption of phase immiscibility. Consequently, we could define the initial volume of the hydrogel as 0.5 µL prior to any swelling or drying. Furthermore, Figure 7 illustrates that in the alginate-first configuration, an increase in the volume ratio correlated with an increase in hydrogel diameter, while simultaneously decreasing the thickness of both the outer rings and the central region.

**Figure 8:**
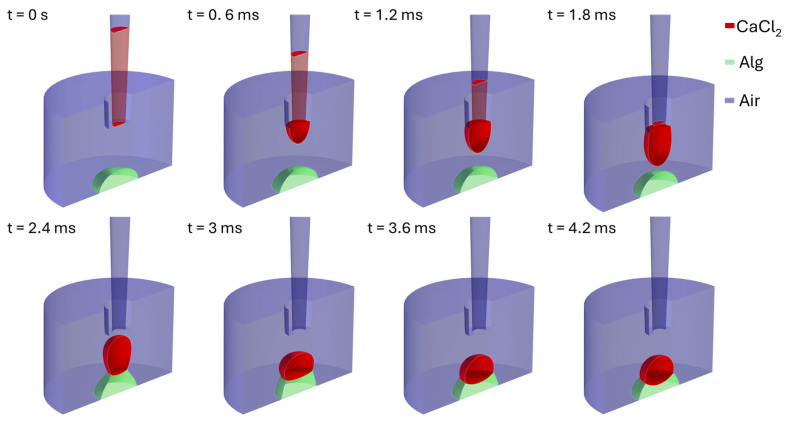
Time-lapse visualization of droplet interactions, showing the sequence when an alginate solution droplet was spotted before a 0.1 M CaCl_2_ droplet, when R = 1.

Figure 8 illustrates the initial moments of dispensing 0.1 M CaCl_2_ from a pipette tip and the interactions between 0.1 M CaCl_2_ and alginate solutions, where the alginate solution was spotted before the 0.1 M CaCl_2_ with a volume ratio of R = 1.

Table 2 presents the numerically estimated surface area of the alginate phase as a function of its volume ratio. When the alginate solution was spotted prior to 0.1 M CaCl_2_, the surface area was 82.91% larger compared to when the alginate solution was spotted after 0.1 M CaCl_2_ at a volume ratio (R) of 1. Increasing the volume ratio in the alginate-first configuration led to an increase in both diameter and surface area. However, the diameter can be a limiting factor in certain assays, particularly when microscopy-based detection is employed. Therefore, selecting the appropriate volume ratio is crucial, as it directly impacts the diameter of the alginate phase.

According to Table 2, the concave hydrogel disc with R = 1 has a 5.47% larger surface area and a 0.72% larger diameter than the concave hydrogel disc with R = 0.5. Likewise, the concave hydrogel disc with R = 2 shows a 12.80% increase in surface area and a 13.33% increase in diameter relative to R = 1. In both comparisons, R was doubled, yet the change in dissolution time is not symmetric: the difference between R = 1 and R = 0.5 is 22 s, whereas between R = 2 and R = 1, it is 7 s. In the first comparison, the percentage increase in diameter is ~0.13× the percentage increase in surface area; in the second, the percentage increase in diameter is ~1.04× the percentage increase in surface area. This pattern explains why the improvement in dissolving time from R = 1 to R = 2 is smaller than from R = 0.5 to R = 1. A larger diameter promotes stronger adhesion between the hydrogel and the polystyrene surface. As a result, a larger disc detaches less readily, and separation of the hydrogel from the surface becomes more difficult.

## 4. Conclusions

In this study, we numerically and experimentally investigated the interfacial dynamics controlling the formation of concave hydrogel discs using a simple deposition technique. Our objective was to establish a predictive method for fabricating hydrogels with enhanced surface area to improve biosensing performance. By integrating experimental methods, including confocal microscopy, with a three-phase numerical model, we successfully simulated the droplet interaction and final geometry of alginate hydrogels. The model was validated by first experimentally determining the contact angles of the solutions and then calibrating the unknown interfacial tension (0.0747 N/m) against 3D confocal images of the fabricated structures.

The three-phase flow model successfully predicted hydrogel geometries with reasonable accuracy, validating the assumption of phase immiscibility during crosslinking. Therefore, we assumed that the initial volume of hydrogel is equal to the volume of alginate solution. The numerical analysis quantified the significant advantage of the concave structure, which possesses an 83% greater surface area than a hemispherical disc of the same volume ratio (R = 1). The model demonstrated that increasing the volume ratio of CaCl_2_ to alginate steadily increased the disc’s diameter and total surface area, though diameter expansion may impose constraints in microscopy-based assays. We showed that increasing the CaCl_2_ to alginate volume ratio steadily increased dissolution speed. Gains in surface area accelerated EDTA access, while simultaneous growth in diameter increased adhesion; this tradeoff explains the small improvement between R = 1 and R = 2 despite the larger surface area.

The ability to control surface area through simple parameter adjustments provides a versatile platform for enhancing various bioassay formats. These results demonstrated that reaction speed in a fixed amount of hydrogel can be controlled by adjusting the volume of CaCl_2_ solution. This works because changing the CaCl_2_ solution volume altered the hydrogel’s surface area without affecting its total volume. Future work should explore the relationship between surface area enhancement and actual biosensor performance metrics, including detection limits and reaction speed.

## Figures and Tables

**Table 1:** Comparison of numerical and experimental dimensions of hydrogel discs.

Configuration	Volume Ratio	Minimum Thickness (µm)			Disc Diameter (µm)			Average Ring Thickness (µm)			Maximum Height (µm)		
		E	N	% Error	E	N	% Error	E	N	% Error	E	N	% Error
CaCl_2_-first	1	_	_	_	959	900	6.15	_	_	_	301	408	35.55
Alginate-first	0.5	238	240	0.84	1341	1370	2.16	330	412	24.85	_	_	_
Alginate-first	1	168	165	1.79	1367	1380	0.95	322	342	6.21	_	_	_
Alginate-first	2	93	80	13.98	1681	1564	6.96	286	270	5.59	_	_	_

**Table 2:** Numerically estimated surface area of the alginate phase as a function of volume ratio.

Configuration	Volume Ratio	Surface Area (mm^2^)	Diameter (µm)
CaCl_2_-first	1	1.58	900
Alginate-first	0.5	2.74	1370
Alginate-first	1	2.89	1380
Alginate-first	1.5	3.02	1462
Alginate-first	2	3.26	1564
Alginate-first	2.5	3.37	1624
Alginate-first	3	3.56	1706

## Data Availability

Data is contained within the article or Supplementary Material.

## Supplementary Materials

The following supporting information can be downloaded at https://www.mdpi.com/article/10.3390/polym17172341/s1, Video S1: Time-dependent comparison of how concave versus hemispherical magnetically responsive hydrogel discs dissolve in an EDTA solution.
